# Widespread reduction of ozone extremes in storylines of future climate

**DOI:** 10.1038/s44407-025-00019-4

**Published:** 2025-08-04

**Authors:** Tamara Emmerichs, Domenico Taraborrelli, Fuzhen Shen, Sergey Gromov, Michaela I. Hegglin, Andreas Wahner

**Affiliations:** 1 Institute of Climate and Energy Systems: Troposphere (ICE-3), Forschungszentrum Jülich GmbH, Jülich, Germany; 2 Center for Advanced Simulation and Analytics (CASA), Forschungszentrum Jülich GmbH, Jülich, Germany; 3 Institute of Climate and Energy Systems: Stratosphere (ICE-4), Forschungszentrum Jülich GmbH, Jülich, Germany; 4 Max Planck Institute for Chemistry, Mainz, Germany; 5 Department of Meteorology, University of Reading, Reading, UK; 6 Institute for Atmospheric and Environmental Research, University of Wuppertal, Wuppertal, Germany; 7 Present Address: Max-Planck Institute for Meteorology (MPI-M), Hamburg, Germany

**Keywords:** Atmospheric chemistry, Abiotic, Climate and Earth system modelling, Climate-change impacts, Atmospheric science, Climate change, Chemistry

## Abstract

High ozone levels harm people and the environment, especially during extreme weather. Climate change is expected to increase the frequency and intensity of these events, exacerbating vegetation-atmosphere interactions. However, current models predict inconsistent responses to warming, potentially due to simplified vegetation representations. We address this uncertainty by incorporating realistic vegetation responses to abiotic stresses into a global atmospheric chemistry model. By constructing storylines of future climate with fixed anthropogenic emissions, we quantify how temperature and humidity changes affect ozone and associated mortality. Here, we show that locally, vegetation and photochemistry often act in concert to amplify ozone pollution extremes, while increased humidity in the free troposphere tends to suppress background ozone levels. The latter effect becomes more dominant with increasing temperatures, leading to a widespread decrease in ozone pollution across the Northern hemisphere. The storyline approach is an effective method for disentangling drivers of air pollution perturbed by climate change.

## Introduction

Tropospheric ozone (O_3_) has adverse effects on human health^1^ and damages vegetation, reducing ecosystem productivity and crop yields^2^. As a photochemically produced pollutant, O_3_ is also a primary driver of the atmosphere’s oxidation capacity^3^. The regional distribution of ground-level O_3_ is shaped by precursor emissions, vegetation uptake, and photochemistry, all of which depend on meteorological conditions^4^.

Over continental regions, local O_3_ levels typically show a positive correlation with temperature^5^, which is generally well represented in models^6,7^. However, correlations with humidity vary by location, depending on local chemical environments and land-atmosphere coupling regimes^8^. Additionally, drought stress affects the dry deposition of gases like O_3_ on vegetation, which remains challenging for some models to represent accurately^7,9^. Also, biogenic emissions of O_3_ precursors are affected by drought conditions^10^ and require incorporation of soil moisture^11^ or photosynthetic activity^12^ for more accurate modelling. Consequently, most Earth system models (ESMs) still lack a consistent representation of land-atmosphere-chemistry interactions, which is key to predict continental ground-level O_3_ under climate change^13^.

Assessments of future O_3_ pollution usually suggest a climate penalty over populated regions^14^. Such studies have relied on probabilistic (risk-based) approaches using ensembles of model simulations. However, this method is limited by uncertainties in the atmospheric circulation response to climate change^15^, such as shifts in the polar jet stream and associated precipitation events. Instead, we use a physical climate event storyline approach to explore how a specific warming level would affect air pollution under the same atmospheric circulation patterns like the ones observed during recent heatwaves. This approach separates thermodynamic effects from circulation changes^16^. By nudging the model only toward fields of divergence and vorticity from atmospheric reanalysis data^17^, we can reproduce large-scale circulation patterns conducive of recent extreme events and assess Earth system responses under a plausible increased warming^18,19^. To reduce uncertainty and maintain focus, anthropogenic emissions of O_3_ precursors are held constant.

During the summers of 2018, 2019, and 2020, Europe experienced a series of exceptional heat waves. The intensity, persistence, and spatial extent of the 2018 summer heatwave was comparable to the 2003 ‘mega-heatwave’^20^. In 2019, Europe recorded its highest ever temperatures, while 2020 was one of the three warmest years on record^21,22^. In addition, the occurrence of severe droughts in 2018 was not limited to central Europe, but extended to large parts of the Northern Hemisphere (NH)^20^. The severity of Europe’s 2018 heatwave was amplified by soil-moisture feedbacks^23^, which are known to intensify heatwaves^24^. Concurrently, vegetation feedbacks in the region exacerbated extreme ozone pollution^25^.

This study presents, for the first time, physical climate event storylines for air pollution under varying levels of warming, using the global atmospheric chemistry-climate model EMAC. We conduct simulations for 2018–2020 at one *factual* (*+1.1K*) and two sensitivity warming levels (*+2K*, *+2.75K*) relative to pre-industrial conditions. These warming levels represent anomalies in the global mean 2m temperature with respect to pre-industrial values. The simulations separate thermodynamic and dynamic aspects of climate change^18,19^, with thermodynamic variables (temperature, transpiration, moisture) evolving freely, while dynamic variables (atmospheric vorticity and divergence defining the large-scale circulation) are prescribed. This allows us to isolate the thermodynamic effects of climate change on O_3_ extremes, minimising influences from changing weather patterns or stratosphere-troposphere exchange.

Our findings of how ground-level ozone changes in two storylines of future climate are presented in the next section. We then examine the drivers of future O_3_ extremes, reassess the O_3_-climate penalty, and contrast our results with recent literature. Additionally, we analyse changes in various O_3_ pollution metrics across our storylines and assess their impact on human health and terrestrial vegetation. The paper concludes with summarising key findings, including a discussion of the storyline approach and the role of vegetation.

## Results

### Ozone in warmer climates

**Fig. 1:**
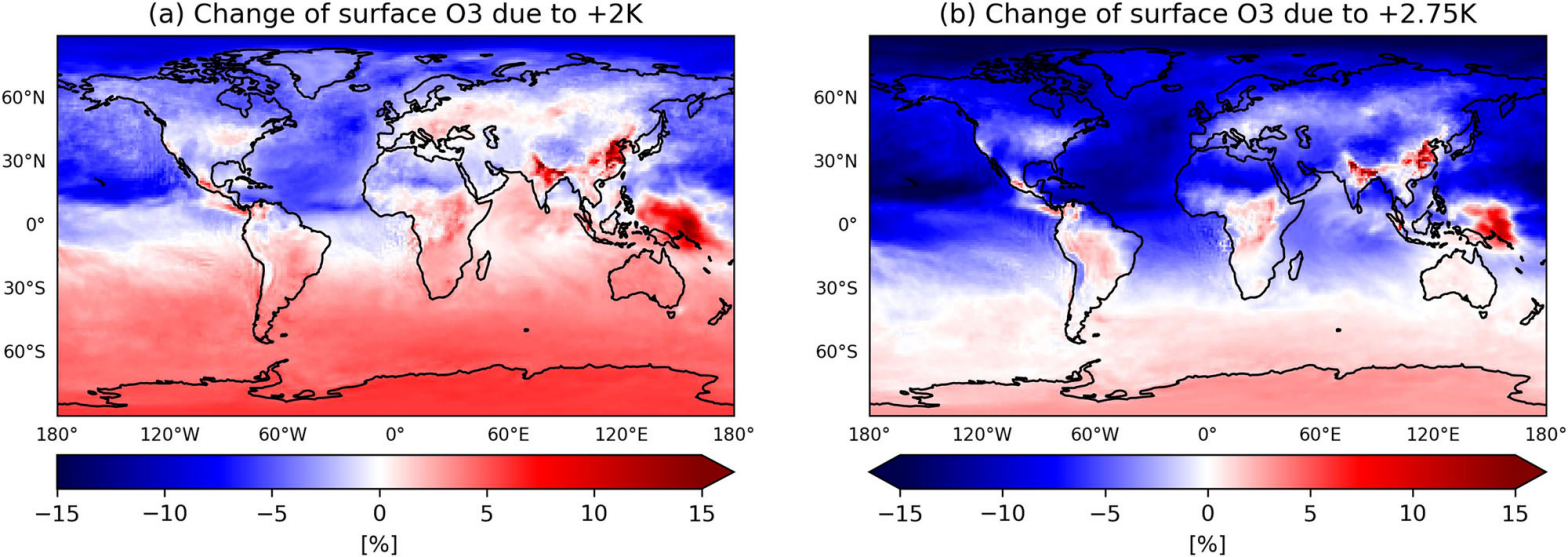
Relative difference of daily (24-h) mean ground-level ozone over the 2018–2020 period. Two scenarios (**a**: *+2K* vs. *factual*; **b**: *+2.75K* vs. *factual*) are shown. Warm and cold colours represent positive and negative changes (NH summer: JJA), respectively.

The 2018–2020 event storyline of a *+2K* climate (Fig. 1) show an average 5–10 % decrease of ground-level O_3_ over the NH oceans and most NH continents due to changes in background O_3_. Over industrial NH regions and throughout the Southern Hemisphere (SH), an O_3_ increase of 5–10% occurs, with regional hotspots in India and China where changes can exceed +10%. However, the highest relative O_3_ increases occurring in the equatorial Pacific Ocean (globally) are due to the local O_3_ minimum in this region, i.e. small baseline values amplify the absolute change (ref. ^26^, Supplementary Fig. S1).

In the *+2.75K* world, the O_3_ reductions across the NH become more pronounced, with decreases up to 15 % over the oceans, also extending into the equatorial region of the SH. The only exceptions to the widespread O_3_ decreases in the NH are found in the most-populated regions of India and China. There, O_3_ increases found for the *+2K* world (Fig. 1a) persist, or only slightly decrease in an even warmer (*+2.75K*) world (Fig. 1b). On average, these changes exceed the internal model variability of daily O_3_ (standardised mean difference: Supplementary Figs. S2, S3, S4) and the results are robust across the 3 years. While the SH O_3_ shows significant increases at *+2K*, these changes are smaller at *+2.75K* (around 5 %), with positive values occurring uniformly across the extra-tropics but confined to land areas in the equatorial region.

Globally, the *+2.75K* warming results in a reduction of the tropospheric ozone burden by about 20 Tg relative to the *factual* climate (371 Tg in 2018, Supplementary Tab. S1). This is in apparent contrast to the analysis of CMIP6 climate projections suggesting an increase in ozone burden due to changes in anthropogenic pollutant emissions and inflow from the lower stratosphere^27^. These effects are essentially excluded by design in our simulations. The storyline approach we use here allows to disentangle these and other drivers of ozone change in the future. In this framework, we conduct a targeted analysis of the role of vegetation and thermodynamic climate feedbacks for ground-level ozone.

### Drivers of ground-level ozone

Ground-level O_3_ in the two warmer climates is a result of the (changing) contributions of (1) photochemistry, and (2) dry deposition and (3) entrainment of free tropospheric air in the boundary layer. While the latter depends on the background O_3_ levels, the first two processes act locally on O_3_ at sub-daily scale. We therefore analyze the daily accumulated values of dry deposition flux and net chemical tendency of ground-level O_3_. The spatial distribution of the respective changes remains consistent for the analysed years, underscoring the robustness of the underlying physical and chemical processes.

**Fig. 2:**
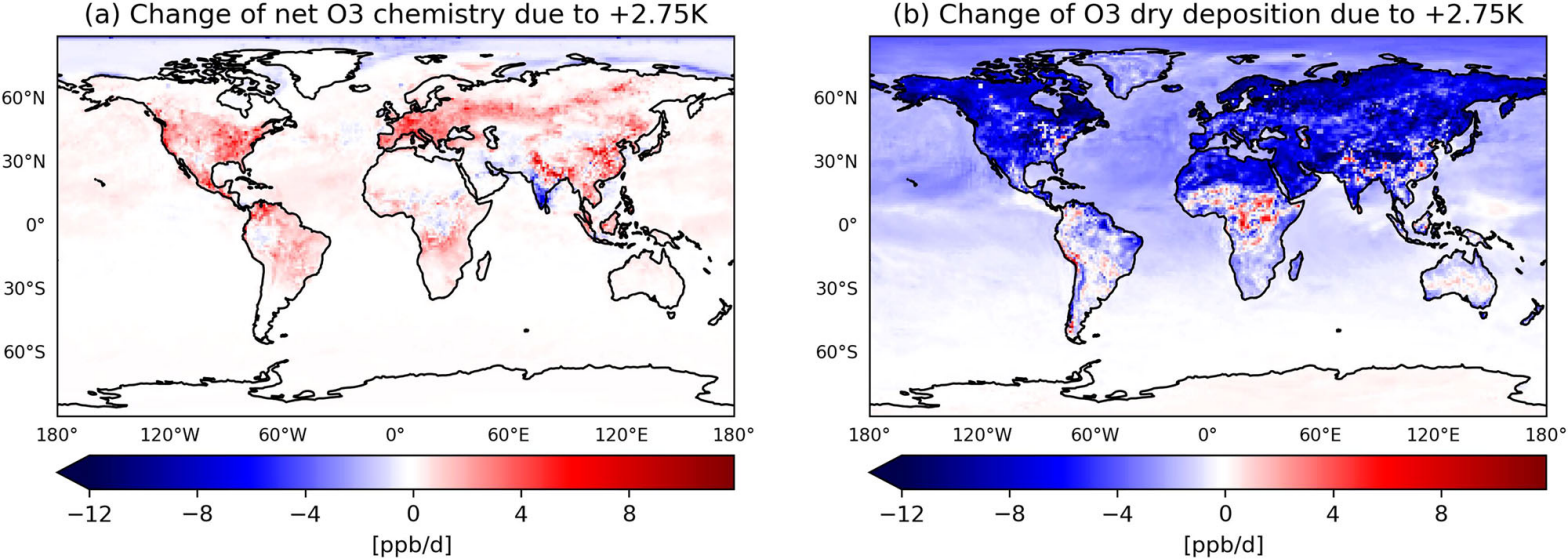
Absolute difference of O_3_ terms during NH summer (JJA) 2018. **a** The daily accumulated net O_3_ chemistry at ground level and **b** the O_3_ dry deposition between the *+2.75K* and the *factual* climates. Warm and cold colours represent positive and negative changes, respectively.

**Fig. 3:**
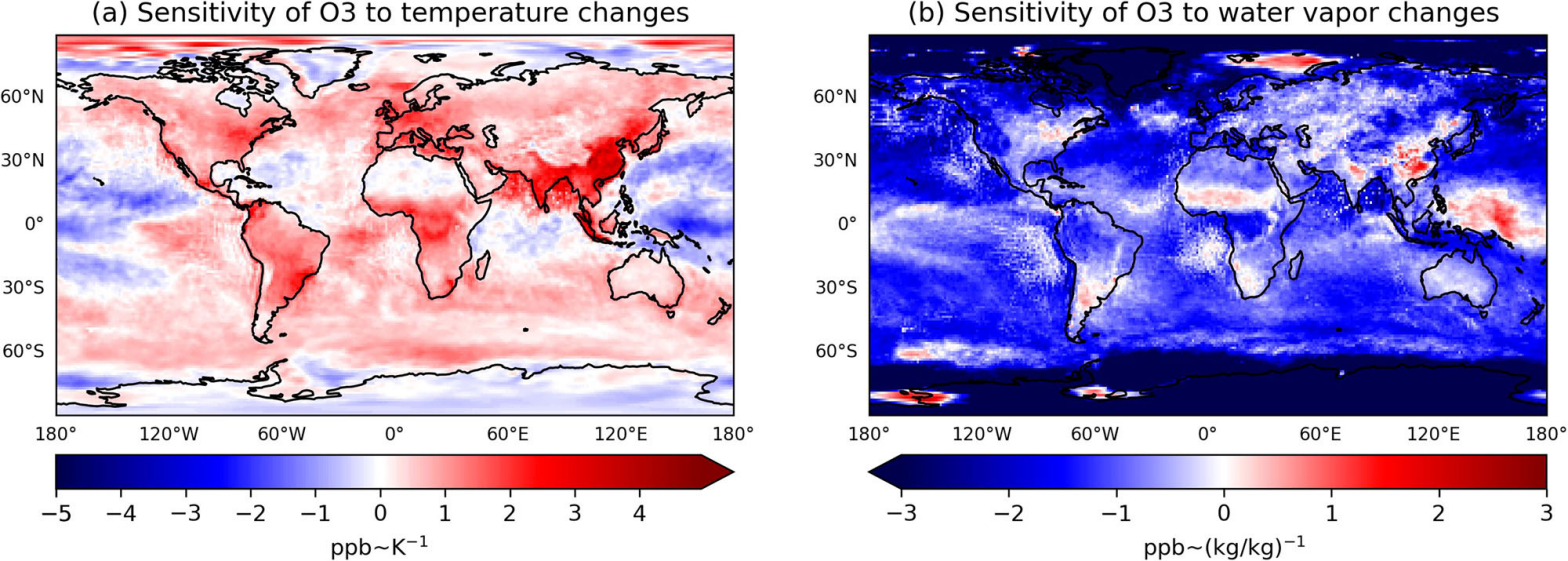
Sensitivity of daily mean ground-level O_3_ to the warming from the *factual* to the *+2.75K* climate over the 2018–2020 period. The spatial distribution of linear regression slopes of **a** O_3_ vs. temperature changes (dO_3_/dT in [ppb/K]) and **b** O_3_ vs. water vapour changes (dO_3_/dH_2_O [ppb/(kg/kg)]). Warm and cold colours represent the positive and negative O_3_ mixing ratio sensitivities, respectively.

In the *factual* climate, ground-level O_3_ production exceeds the chemical loss in most continental regions. In warmer climates, local drivers push towards increased ground-level O_3_ concentrations, particularly in the eastern US and East Asia. For instance, in the *+2.75K* storyline, boreal summer net chemical production increases by up to 10 ppb/d in these regions (see Fig. 2a). Higher temperatures enhance the radical chemistry, as reflected in the dO_3_/dT slope (Fig. 3a), favouring O_3_ production. At the same time, elevated HO_2_ and OH levels also increase chemical loss, but the net effect remains an overall O_3_ increase. The availability of radicals depends on biogenic VOCs and NO_*x*_, which together largely control the levels and cycling of HO_2_ and OH. In our storyline approach, it is mainly the variations in plant emissions—driven by drought stress, CO_2_-inhibition effects, and temperature changes—that influence the chemical environment (see ‘Methods’). In addition, NO emissions from soil influence the chemical regime, particularly in remote continental environments where they modulate O_3_ production efficiency. The notable exception with a strong decrease in O_3_ chemical production is over Southern India affected by the summer monsoon. Upon warming, intensified precipitation enhances scavenging of ozone and its precursors^28^.

Enhanced O_3_ levels in central and eastern Europe are linked to increased isoprene emissions from plants (Supplementary Fig. S5). This is consistent with earlier findings^5^. Under high-NO conditions, stronger isoprene emissions boosts peroxy radical and consequently O_3_ formation. However, under low-NO conditions such as above pristine tropical forests, isoprene emissions primarily contribute to O_3_ loss, except in regions where increased CO_2_ inhibits VOC emissions^29^.

While daytime O_3_ production is enhanced in high-NO_*x*_ regions due to increased isoprene-driven radical chemistry, nighttime O_3_ loss is also strengthened in a warmer climate due to increased soil NO emissions. During the night, nitrogen oxides (NO) emitted from the soil consume O_3_ to form NO_2_ and NO_3_, eventually producing HNO_3_ which is efficiently taken up by vegetation and cannot contribute to daytime ozone production. In the *+2.75K* world, this stronger nighttime loss reduces the O_3_ level relative to the *factual* world, particularly in the SH, where it compensates for up to 20% of the daily O_3_ changes (Supplementary Fig. S6). This compensating effect is less pronounced in the NH (other NO sources are more important). Globally, soil NO emissions are calculated to increase by about 0.7 Tg(N)/yr (≈10%) in the future.

While changes in chemical production and loss shape O_3_ concentrations in a warmer climate, surface processes such as dry deposition and, specifically, plant uptake also play a crucial role in determining regional O_3_ levels. The interactions between vegetation, soil moisture, and atmospheric composition modulate O_3_ deposition patterns. In particular, future CO_2_ increases reduce plant stomatal opening in regions like the Amazon, Central Africa, and Southeast Asia, leading to reduced O_3_ uptake by vegetation. Conversely, higher humidity enhances the O_3_ uptake at leaf surfaces^30^.

In the *+2.75K* storyline, O_3_ deposition decreases in NH boreal forests. This reduction is primarily explained by increased O_3_-induced stress, which causes 20–30% more damage to vegetation, limiting its ability to take up O_3_. Additionally, drought stress increases of 5–10%, resulting from higher temperatures^9^, further weakens plant health and increases vulnerability to O_3_ exposure. Changes in soil moisture in warmer climates are mostly localised. There is no regional pattern of change in the land-atmosphere coupling regimes and hence in dry deposition velocity. Overall, the change in the actual dry deposition flux (Fig. 2b) is dominated by the changes in the O_3_ concentrations (Fig. 1b).

**Fig. 4:**
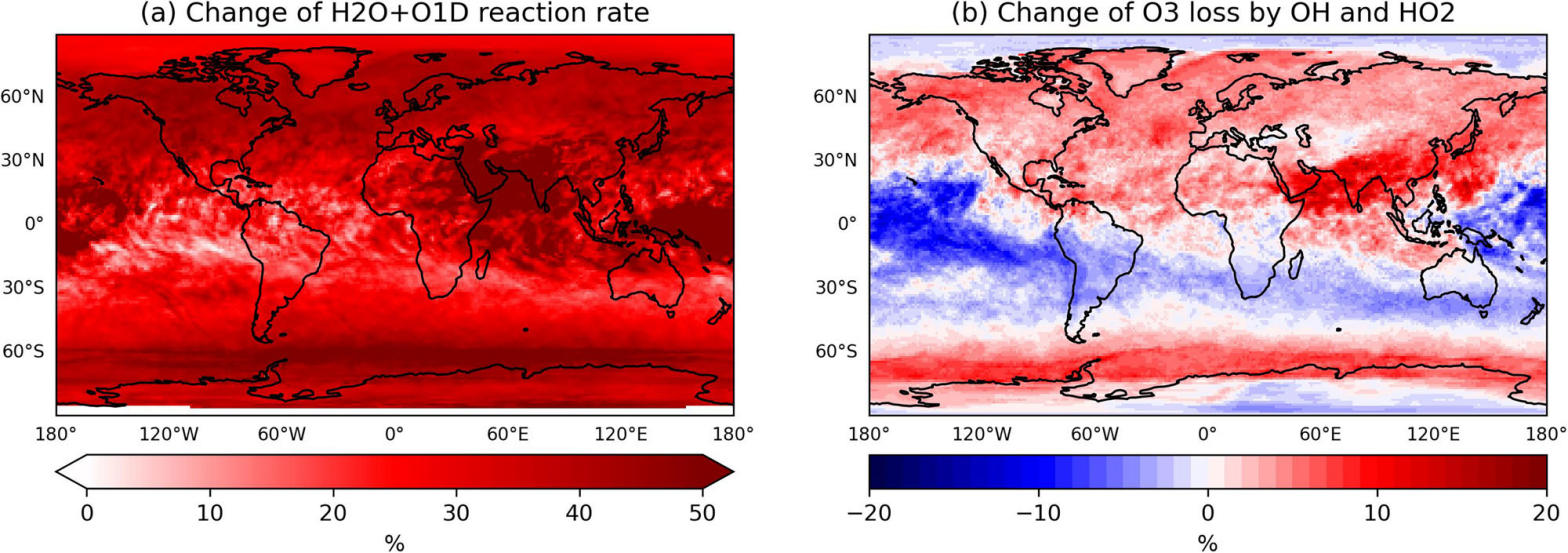
Major O_3_ losses in the free troposphere. Relative change of the (daily accumulated) **a** *O*(^1^*D*)+*H*_2_*O* reaction rate and **b** *O*_3_ loss by OH and *HO*_2_ ≈ 8 km above the surface due to the *+2.75K*-climate during summer (JJA) 2018.

In addition to local drivers, entrainment of background O_3_ from the free troposphere is also an important contributor to ground-level O_3_. Background ozone is strongly reduced in the *+2.75K* storyline simulation (Fig. 1b). The changes are most pronounced over the oceans and affect the continents via long-range transport, e.g. from the North Atlantic to Northern Europe^31^. Much of the decrease is due to enhanced O_3_ destruction in the free troposphere (Fig. 4), where, in contrast to the (continental) boundary layer, water vapour is the limiting factor for the O(^1^D) loss^32^. This (tropospheric) loss term increases by 11.7 % (150 Tg/yr) in the NH and by 6.1% (154 Tg/yr) globally, consistent with findings of widespread decreases in O_3_ in warmer and moister climates^13,33,34^.

The overall changes in ground-level O_3_ result from the interplay between local drivers (i.e. photochemistry, precursor emissions, and dry deposition) and reductions in background O_3_. The balance of these competing processes varies regionally, highlighting the complexity of ozone-climate interactions.

### Ozone-climate penalty or benefit?

The ‘O_3_-climate penalty’ refers to the amplification of O_3_ air pollution due to global warming, quantified as the rate of O_3_ change per unit of temperature change. Several methods for estimating this penalty exist in the literature, such as the long-term correlations between observed O_3_ and temperature, or perturbation analyses of temperature changes (ref. ^35^ and references therein). Here, we estimate this effect using the *factual* EMAC and the *+2.75K* storyline simulations (Fig. 3).

The sensitivity of O_3_ to temperature changes (dO_3_/dT) increases significantly, reaching a maximum of +5 ppb/1K in East and South Asia (Fig. 3a). This is consistent with the results by Zanis et al.^13^, which attributes the enhanced sensitivity to stronger anthropogenic NO_*x*_ emissions. In contrast to their study, our simulations with fixed anthropogenic emissions indicate that the increase in dO_3_/dT at ground level is related to enhanced biogenic VOC emissions in response to warming. Indeed, this relationship has already been observed in China^36^. The regional increase of O_3_ with temperature in the North-East of the US (positive slopes, Fig. 3a), aligns with observations in this region^7^. Over the oceans, we find negative slopes only in the tropical Pacific, in agreement with the CMIP6 model ensemble under the ssp370 scenario^13^.

Future increases of atmospheric water vapour particularly affect the chemistry in the free troposphere^32^. The sensitivity of O_3_ to humidity (dO_3_/dH_2_O, Fig. 3b) over North Eastern America shows a north-south gradient going from positive to slightly negative values. This is related to a transition of the land-atmosphere coupling from a soil water-limited to a energy-limited regime^8^. Since these coupling regimes do not change for North America in the *+2K* and *+2.75K* scenarios, the changes in ground-level O_3_ are consistent with the dO_3_/dH_2_O reported by Kavassalis et al.^7^. A similar reason also applies to East Asia, where positive slopes reflect the shift from a VOC-limited to a NO_*x*_-limited chemical regime^37^. The concurrent increase of O_3_ and H_2_O in the tropical West Pacific Ocean is related to strengthening moist convection, which draws humid, O_3_-rich air towards regions with low tropospheric O_3_ columns^38^.

While our assessment of future O_3_ sensitivities confirms a significant increase in O_3_ production with rising temperature—the so-called ‘climate penalty’—here we highlight in addition the importance of the O_3_ sensitivity to water vapour. In most regions of the NH with moderate NO_*x*_ pollution, this effect may result in a regional ‘climate benefit’ by counteracting the temperature-driven O_3_ increase.

### Ozone pollution extremes

**Fig. 5:**
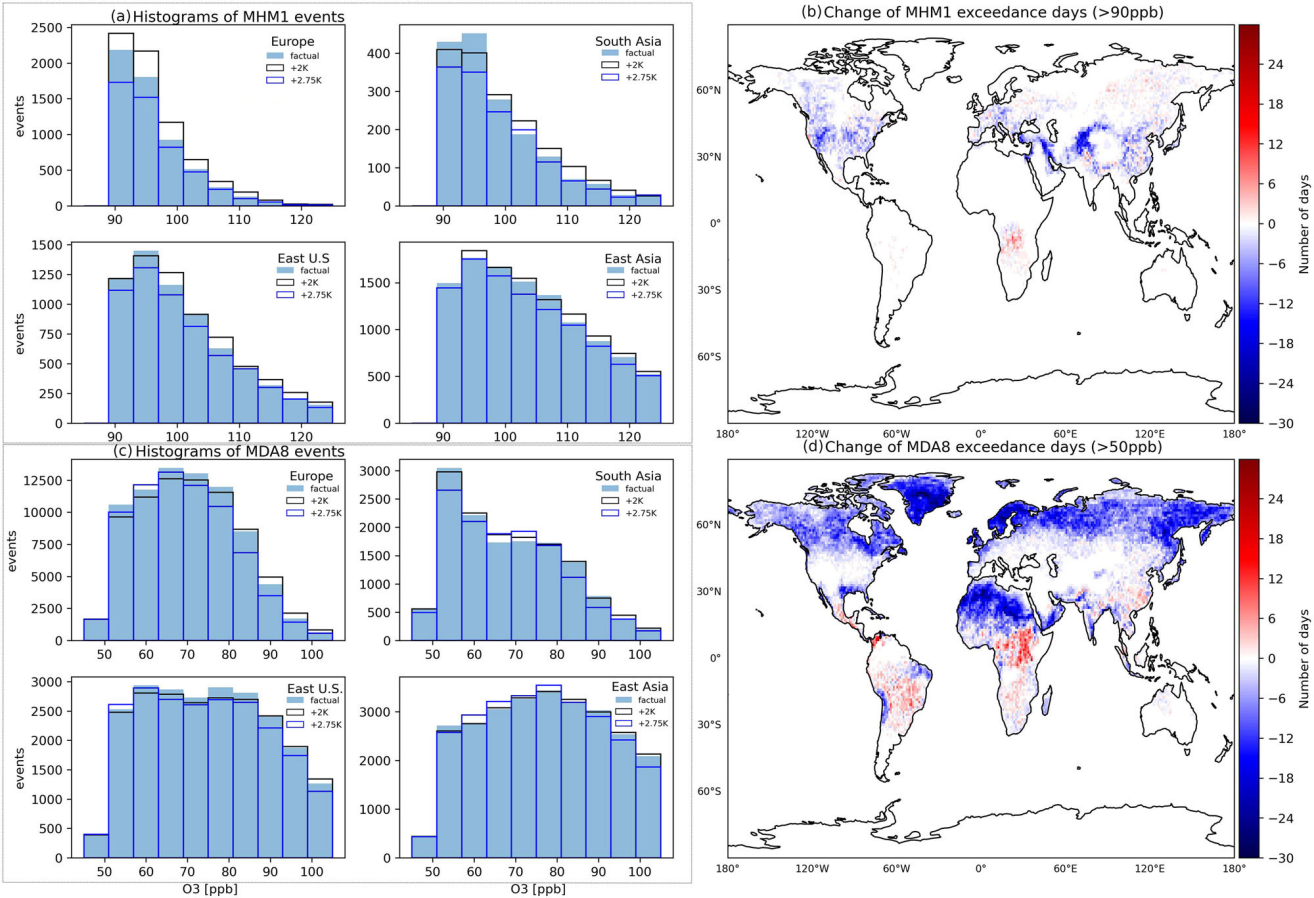
O_3_ pollution metrics in summer 2018. Number of events for **a** maximum daily 1-h average ground-level O_3_ exceeding 90 ppb (MHM1) and **c** daily maximum 8-h running average (MDA8) exceeding 50 ppb in different regions and the corresponding global distribution of the different number of days for **b** MHM1 and **d** MDA8 between *+2.75K* and *factual* climates. Dark blue solid, black hollow and blue hollow bars represent *factual*, *+2K* and *+2.75K* climates, respectively. The warm and cold colours in the colour bar represent positive and negative changes, respectively. Note that the regions are defined according to the sixth IPCC assessment report^84^: Europe (16,17,18, only land), S.E. U.S: 5, East Asia: 35, South Asia (SAS): 37.

We now consider the implications of the simulated O_3_ changes for human and ecosystem health in the two storyline experiments. Assessing these impacts requires robust metrics to characterise O_3_ extremes, two of which are used here (Fig. 5). While the daily maximum 1-h O_3_ mixing ratio (MHM1O_3_) captures the short-term variability due to chemical ozone production and loss, governments often refer to the daily maximum 8-h running average of O_3_ mixing ratio (MDA8O_3_, e.g. the World Health Organisation guidelines^39^, 50 ppb threshold) as a guideline. MDA8O_3_ takes into account the relevant human exposure time to O_3_ and is influenced by factors such as dry deposition^1^.

Under present-day conditions (equivalent to a *+1.1K* warming relative to pre-industrial times, the *factual* simulation), the highest number of MHM1O_3_ events exceeding 90 ppb falls within the 90–100 ppb range across the studied regions: Europe, South Asia, East U.S. and East Asia (first four histograms, Fig. 5a). East Asia experiences the most extreme events, with a maximum of 1750 occurrences. In most of these regions, the number of MHM1O_3_ extremes decreases exponentially as the threshold increases beyond 90 ppb, following a log-normal distribution up to 125 ppb. A significantly higher number of MDA8O_3_ than MHM1O_3_ events occur in Europe (Fig. 5c), indicating the important role of long-range transport, especially in summer when Asian and North American emissions contribute more to European O_3_ levels than regional emissions^31^.

In the *+2K* storyline, the number of MHM1O_3_ events increases across all four regions (Fig. 5a, grey lines). By far the largest increase in number of extreme events (about 800, 13%) is seen in Europe compared to changes of less than 6% in the US and Asia. These regional differences indicate that higher baseline O_3_ levels in Europe due to the European heatwaves in summer 2018 is more prompted for increases in O_3_ extremes. MDA8O_3_ extremes intensify in Europe and Asia, occurring only at high O_3_ levels in Europe and at medium levels in South Asia due to the different background pollution and chemistry regimes (Fig. 5c, grey lines).

As the world continues to warm, the O_3_ extremes become less frequent in the NH due to a decline in background ozone transported from distant regions. However, in the Tropics and Subtropics this effect is largely offset by an increase in net O_3_ production, as most evident in Fig. 5d. In regions like South China, with high-NO and a VOC-limited ozone regime, the strong net O_3_ production is fuelled by biogenic VOC emissions especially in summer^40^. Enhanced isoprene emissions as predicted in the two warming scenarios (see Supplementary Fig. S5) further drive O_3_ production up that cannot be completely offset by the entrainment of air with lower background O_3_.

### Detrimental effects on humans and plants

Finally, we estimate how structural changes in O_3_ pollution would affect plants and human health in the *+2K* and *+2.75K* storylines, given the same atmospheric circulation as observed in 2018–2020. Note that these impacts are derived for constant anthropogenic pollutant emissions to reduce a key driver of uncertainty in such calculations.

**Fig. 6:**
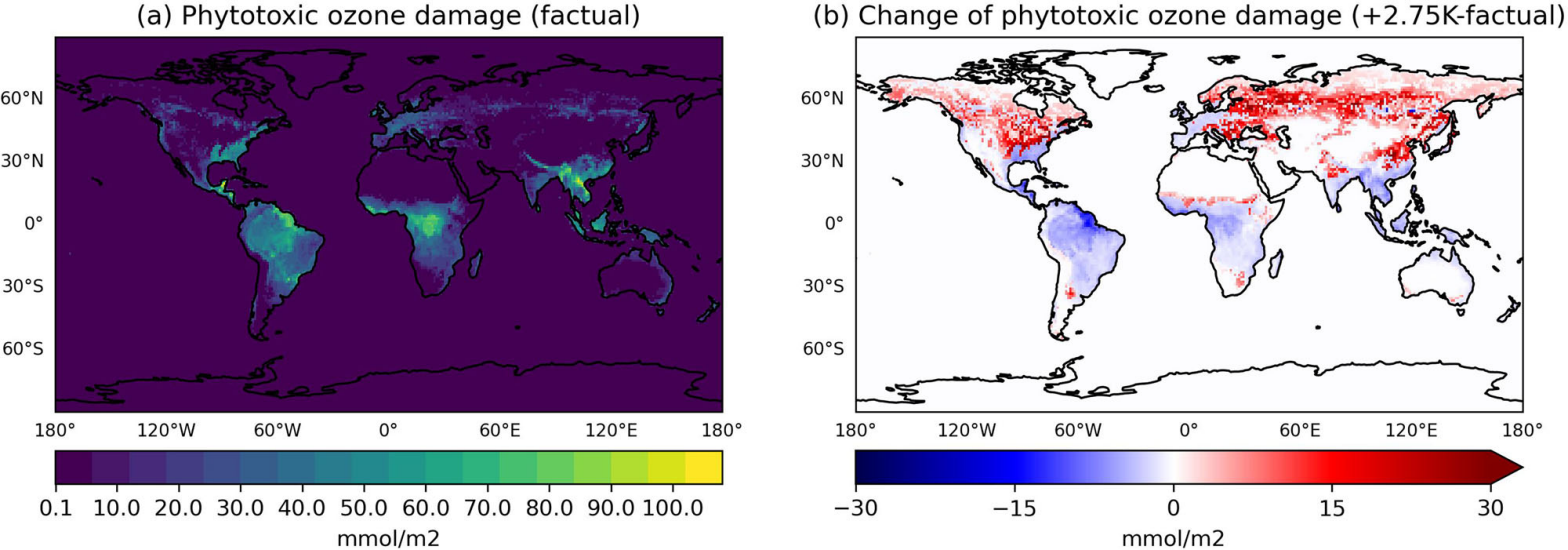
Mean phytotoxic ozone damage over the 2018–2020 period. **a** *factual* climate (+1.1K vs. preindustrial) and **b** absolute difference between the *+2.75K* climate and *factual* climate. Warm and cold colours in the colour bar represent **a** maximum and minimum values and **b** positive and negative changes, respectively.

We first assess the cumulative stomatal O_3_ uptake using a dynamic threshold (see ‘Methods’), referred to as the phytotoxic ozone damage (POD). Our estimated global distribution, with local values reaching 100 mmol(O_3_)/m^2^, is generally in agreement with the estimates by^41–43^. The highest POD values consistently occur over tropical forests, although they are somewhat overestimated due to a differences between plant types used in our model and those in reality. In the *+2.75K* storyline, POD significantly increases in NH forests (Fig. 6b), despite an overall decline in summer O_3_ levels. This increase in stomatal O_3_ fluxes is primarily driven by reduced atmospheric stability, which enhances aerodynamic transport to vegetation and the overall deposition velocity^44^.

**Fig. 7:**
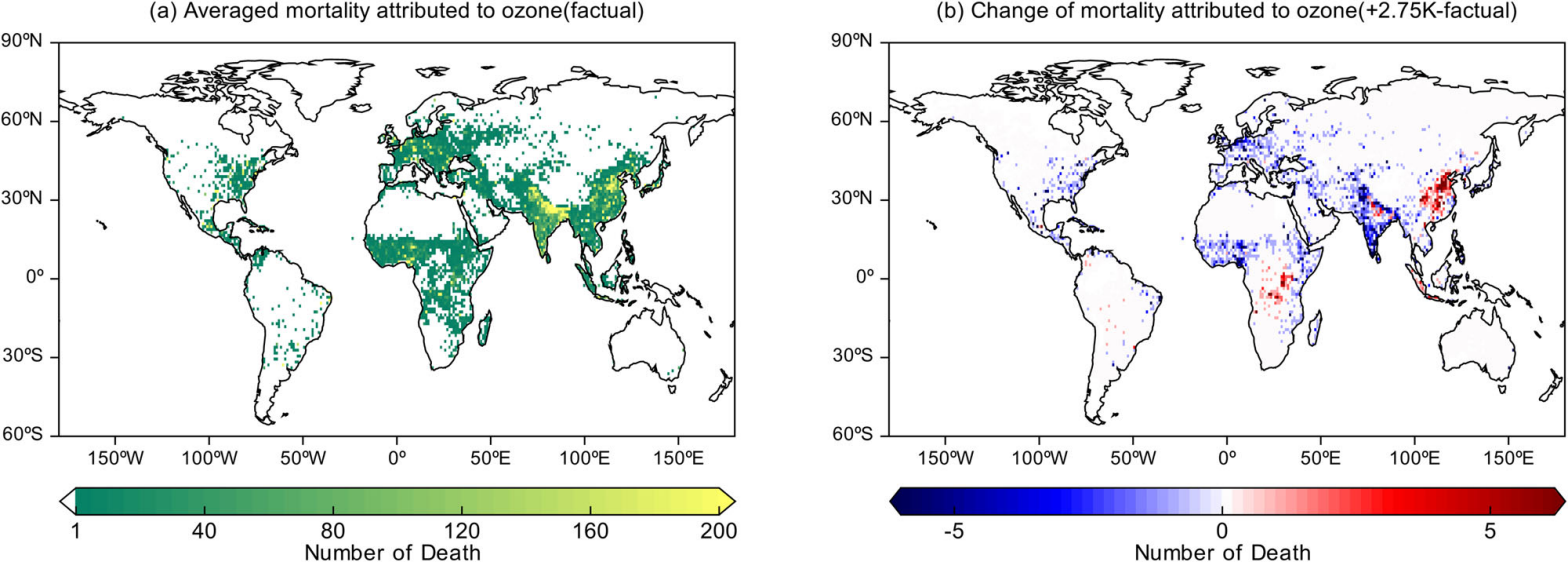
Mean premature mortality over the years 2018–2020. The global distribution of **a** mean premature mortality in *factual* climate (+1.1K vs. preindustrial) and **b** difference of premature mortality between *+2.75K* and *factual* climates. (Note: white colour in colour bar (**a**) means zero, warm and cold colours represent the positive and negative changes, respectively).

We also assess human health impacts by estimating premature mortality due to long-term exposure to elevated O_3_ levels (see ‘Methods’). Based on Fig. 7a, the estimated annual total is at 0.14 million O_3_-attributable mortality cases, primarily concentrated in China and India, two of the world’s most polluted and densely populated regions. Comparisons with other studies show higher global estimates for 2019 (with 0.42 million deaths from O_3_-attributable chronic respiratory disease and 0.37 million deaths from chronic obstructive pulmonary disease)^45,46^. These discrepancies likely stem from methodological differences, including the use of a lower relative risk for all-cause mortality in this study (1.01 vs. 1.06 relative risk,^47^) and the use of different O_3_ thresholds^48^. Additionally, our estimates may be lower because we assume fixed (‘present-day’) anthropogenic emissions, while other air climate change studies incorporate future emissions scenarios (e.g. ssp3.70: 3.12 million deaths; RCP8.5: 0.32 million deaths)^49,50^. Relative to the *factual* world, ground-level O_3_ in the *+2K* storyline (Supplementary Fig. S7) leads to a higher mortality worldwide (all-causes, 2018–2020 average). In contrast, in the *+2.75K* storyline, the corresponding number of deaths decreases in most countries, except for China and several African countries (Fig. 7b), due to a widespread decrease in ground-level O_3_ (Fig. 1b). In China, the number of excess deaths increases slightly by 375 across the country, followed by 83 in the Democratic Republic of the Congo, and by 19 in Angola. The differences between the two storylines and the *factual* estimates show that the shift to the warmer climate significantly reduces the global health burden (Supplementary Fig. S7 for *+2K* and Fig. 7b for *+2.75K*). India (the world’s most populated country) benefits the most from the shift to the *+2.75K* storyline, with an estimated 2298 avoided premature deaths (compared to 450 in the USA). Although the number of deaths in China increases under the *+2.75K* storyline relative to the *factual* climate, the rise is smaller than in the *+2K* storyline. Overall, the *+2.75K* storyline prevents 78.2 % of O_3_-related deaths globally, highlighting the substantial health benefits of lower ground-level O_3_ concentrations in this warmer climate scenario.

## Discussion

This study assesses the evolution of ozone pollution in a warmer world by examining two different physical climate event storylines. These storylines are designed to address the question: How would ozone pollution and its impacts under the heatwaves of 2018–2020 have unfolded in a world that is *+2K* and *+2.75K* warmer, assuming the same atmospheric circulation patterns? Using fixed anthropogenic emissions, this methodology provides a unique way to focus on the thermodynamic effects of climate change and its impacts on the short-term vegetation response.

Our results show that ozone extremes in the *+2K* warmer world will increase in many parts (primarily across the SH and in highly populated regions of the NH) due to the amplification of O_3_ production by temperature and higher biogenic emissions. In contrast, a warming of *+2.75K* leads to a dominant increase of the major O_3_ loss terms in the NH, resulting in a substantial reduction of O_3_ extremes across the NH and the SH equatorial regions, in line with some previous studies (e.g. ref. ^13^). Thus, the thermodynamic aspects of global warming above *+2K* indicate (somewhat unexpectedly) potential benefits for air quality, including a reduction of the frequency and intensity of pollution extremes harmful to human and vegetation health. The point of transition from climate penalty to benefit for ground-level ozone over the continents is regionally dependent on many factors like interactions with vegetation and changes in anthropogenic emissions. Narrowing down the warming levels for these transitions would require many storylines differing for much lower degree of warming. Nevertheless, considering the positive bias global models have^51^ and the current air quality policies in Europe and Asia, the onset of ozone-benefit over the continents might happen at a degree of warming much lower than is suggested by *+2.75K* simulation in this study.

Conducting the first storyline-based air pollution study shows its suitability for extending the knowledge gained from classical probabilistic predictions. A significant uncertainty source in model-aided climate change studies, the uncertainty due to changes in atmospheric dynamics, is largely avoided by the storyline approach, as is the use of fixed anthropogenic emissions. The computational effort, in addition, is significantly reduced compared to conventional probabilistic approaches.

The storyline approach offers a very versatile method for the scientific community to explore future changes in air pollution. By incorporating plant sensitivities to drought and ozone, this study enhances the realism of vegetation-atmosphere interactions, which is crucial for reducing uncertainties in future air quality and climate projections over continents. While our results may be sensitive to parameterisations of the relevant processes (e.g. vegetation response, kinetic chemistry scheme, etc.), our work underscores the need to further study weather-composition interactions in a warming climate. The storyline framework presented here should also be expanded to account for changes in land cover, wildfire intensity and frequency, and anthropogenic emissions. Disentangling these effects from the changes in atmospheric circulation will ensure a comprehensive understanding of future changes in air pollution.

## Methods

### Global atmospheric chemistry model

In this study we use the ECHAM/MESSy global atmospheric chemistry model (EMAC), which is based on the fifth-generation European Centre Hamburg general circulation model [ECHAM5, version 5.3.02;^52^] coupled with the Modular Earth Submodel System (MESSy)^53,54^. MESSy provides a flexible infrastructure with more than 30 different submodels representing chemical, physical and biogeochemical processes for coupling processes to build comprehensive ESMs. The gas-phase chemistry is based on a comprehensive kinetic mechanism (310 reactions, 155 species) as used in the EMAC simulations for the Chemistry-Climate Model Intercomparison Project (CCMI2)^55,56^. Due to the complexity of the kinetic chemistry mechanism, the kinetic model is augmented with additional diagnostic of chemical production and loss of compounds of interest^57^. In addition to the conventional odd oxygen family (O_*x*_) approach to estimate atmospheric ozone budget (detailed in Supplement Tab. S1), the production and loss rates of O_3_ in different reactions, as well as total production, loss and net rates, are diagnosed in the simulations. The 7-mode aerosol scheme simulates black carbon, organic matter, dust, sea spray, sulphate and ammonium nitrate aerosols^58^. A simplified scheme for secondary organic aerosols production from major precursors is included^59^.

### Emissions

The anthropogenic emissions are branched off from the EMAC CCMI2 simulation^56^ output for the respective periods. The mixing ratios of long-lived species (greenhouse gases: CO_2_, CH_4_, N_2_O etc.) at the lowest model layer are assimilated via Newtonian relaxation (every 3 h) to the projected mixing ratios branched off from the EMAC CCMI2 simulation output. The lightning emissions of NO are based on the correlation between the convective cloud top height and the occurrence of flashes and tuned to an average total of 1 Tg(N)/yr in order to achieve realistic present-day O_3_ burden in the troposphere. Soil NO emissions are dependent on temperature, precipitation and soil fertilisation and are calculated with the YiengerI*&*Levy algorithm^60^. Global emissions in EMAC are about 7 Tg(N)/yr^61^. The biogenic emissions of isoprene and other hydrocarbons are modelled with the MEGAN algorithm. The model accounts for the CO_2_ inhibition effect using the online calculated CO_2_ concentration within the plants^62^. The unstressed emissions fluxes are first calibrated to best estimates by^63^. Then, a drought stress factor based on the CO_2_ assimilation rate is applied according to ref. ^12^. Final modelled isoprene emissions for the 2018–2020 period are 329 Tg/yr, consistent with^64^.

### Abiotic stresses

The transpiration and dry deposition process at canopy scale is represented by a photosynthesis scheme that describes the CO_2_ assimilation of plants based on physiological considerations (here only for crops) as a function of CO_2_, temperature, radiation, available and humidity as used in the IFS model^65^. Additionally, two abiotic stressors were implemented for the purpose of this study. First, the plant response to drought stress depends here on leaf water potential which have been shown to succeed over the common used soil-moisture stress factor^9,66,67^. The O_3_-induced damage, based on^68^, assesses the O_3_ flux derived from the multiple resistance scheme^69,70^ against the phytotoxic threshold. Different from other studies, we implemented a dynamical measure for this based on the gross assimilation of plants and a proportionality constant of 0.20 μg(O_3_)/mg(CO_2_)^71^. To consider the limited lifetime of leaves we reset the accumulated ozone damage when the vegetation density decreases consecutively for 2 months. Vegetation density is prescribed with a monthly averages of Leaf Area Index (LAI [m^2^/m^2^]) obtained by a Moderate Resolution Imaging Spectroradiometer.

### Storyline approach

A storyline is a narrative description of a scenario(s) focused on the main characteristics and dynamics, and the relationships between key driving forces (IPCC, https://www.ipcc-data.org/guidelines/pages/definitions.html). We follow Shepherd et al.^72^ defining a physical climate event storyline ‘as a physically self-consistent unfolding of past events, or of plausible future events or pathways’. Across the storyline simulations performed here the large-scale dynamics of the recent past (2018–2020) is fixed by nudging only the horizontal winds (via divergence and vorticity) up to a altitude of 12 hPa. Nudging is a Newtonian relaxation of prognostic variables by^17^ towards ERA5 meteorological reanalysis data^73^. It is applied to all wave numbers, up to 106 as the spatial model resolution is T106L47MA (≈1.1° × 1.1°). The relaxation times are between 6 and 48 h. These are numbers higher than the ones used by other studies^18,19^ and were chosen to minimise model deviation from observations. Temperature and pressure are not nudged allowing the model to give a thermodynamic response in the warmer climates. The latter are realised by perturbing the forcing associated with:

(1) the ocean for which synthetic sea surface temperatures (SST) patterns consistent with a 2K and 2.75K higher global average temperature are prescribed^18^. To this end, we used sea and land surface temperature data simulated by the first ten members of the MPI-ESM ssp245 ensemble (2015–2100) for CMIP6^74,75^. The 2m temperature anomaly relative to the pre-industrial period 1850–1920 can be found in the supplement (Fig. S12). Following the approach by^76^, the SSTs at year y and month m for the warmer world scenario (*SST*_*y*,*m*,+2*K*_ and *SST*_*y*,*m*,+2.75*K*_) are calculated by perturbing the SSTs for the *factual* simulation ($SS{T}_{y,m}^{ERA5}$) with temperature anomalies. The latter are obtained by (a) taking the average SSTs respectively for 2064–2073 ($SS{T}_{+2.75K}^{CMIP6}$) and 2090–2100 ($SS{T}_{+2.75K}^{CMIP6}$) relative to the historic period 1850–1920 ($SS{T}_{y,pi}^{CMIP6}$) and (b) applying a weighting factor *w*_*y*_, multiplied with the difference of the future and *pi* SST values. This gives the ‘warming pattern’, which is based on normalised near-surface temperatures of the 1850–2100 period. The weighting factor *w*_*y*_ thereby accounts for the fraction of the total warming (relative to a pre-industrial baseline) that has already occurred by year y since pre-industrial time. It considers both the temporal evolution of warming and the spatial variations to reflect regional differences in warming rates. For the *+2K* scenario the formula is

1 $$SS{T}_{y,m,+2K}=SS{T}_{y,m}^{ERA5}+{w}_{y}(SS{T}_{+2K}^{CMIP6}-SS{T}_{y,pi}^{CMIP6})$$

(2) The long-lived climate gases (LLCG) CO_2_, CH_4_, N_2_O, CFC11, CFC12 are nudged with data branched off from the CCMI2-RD2 simulation performed with EMAC^56^. The RD2 CCMI2 simulations (several members) reach a global average temperature anomaly of 2.75K at the end of this century, following the SSP2-4.5 CMIP6 scenario (https://www.sparc-climate.org/wp-content/uploads/sites/5/2021/07/SPARCnewsletter_Jul2021_web.pdf). Global average time series of LLCG are shown in Supplementary Fig. S13. Anthropogenic emissions of short-lived gases are fixed to the year 2018. The land cover classification is fixed and the leaf area index is prescribed with MODIS data for 2018 (and following years)^77^.

### Ozone bias in the *factual* simulation

The comparison of tropospheric ozone with the chemical reanalysis TES^78^ in Supplementary Fig. S8 (mean of data in 700-800 hPa) shows a mean bias of 10-20 ppb, exemplary for 2018, which is in agreement with the literature. The overestimation over the Indian ocean is due to complex cloud chemistry neglected here^28^. Additionally, high biases occur over mountain ranges (Andes, California, Mongolia) which is linked to the limited model resolution of topography. However, this is a long-standing issue for global models. A comparison of 6 CMIP6 models with ground-level O_3_ observations (TOAR) show a consistent overestimation of 16 ppb in most regions (NH) during the years 2005–2014^79^ similar to^51^. The extremes for ground-level *O*_3_ simulated by EMAC are compared to TOAR data gridded at 1° × 1° spatial resolution. The daily 1 h-max values for 2018 are shown for Europe, China and US (Supplementary Figs. S9, S10, S11). EMAC generally overestimate also the continental ozone extremes in the NH. The biases are larger (up to 10 ppb) in boreal summer. Over China the biases are the smallest. The global burden of 370 Tg estimated by EMAC for the *factual* (+1.1K vs. pre-industrial) climate is within the multi-model estimates (330–380 Tg) from the literature^27,51^. In comparison, EMAC yields about slightly larger chemical production and loss of ozone (see Supplementary Tab. S1). Furthermore, EMAC estimates a lower dry deposition term of 780 Tg/yr (compared to 800–1000 Tg/yr,^27,51^) due to the additional environmental stressors.

### Assessment of premature mortality

We determined the premature mortality number associated with ground-level ozone by applying health impact functions that link variations in air pollution levels to shifts in mortality. The global ground-level ozone concentrations we used are simulated in the EMAC model. Health impact functions for ozone are constructed based on a logarithmic-linear relationship between relative risk (*RR*) and concentrations, which is defined and widely used in epidemiological research^80,81^:

2 $$RR=exp [\beta (C-{C}_{0})],\,C > {C}_{0}$$

where *β* is the concentration-response parameter indicating the additional all non-accidental mortality attributed per unit increase of air pollutant when it is above the threshold concentration. Here, the *β* value for long-term ozone exposure is 1.0% per 10 μg/m^3^ in the peak-season average of daily maximum 8-h mean ozone concentration, which is recommended by Huangfu et al.^47^. *C* is the simulated ground-level ozone concentration and *C*_0_ is the recommended value by the World Health Organization (WHO) air quality guidelines^39^ for long-term exposure to ozone. The attributable fraction (*AF*), which represents the share of the mortality burden associated with the risk factor, was defined as follows:

3 $$AF=(RR-1)/RR$$

4 $$\Delta M=AF\times P\times BMR$$

*AF* yields an estimate for the additional deaths (Δ*M*) when multiplied by the baseline mortality rate (*BMR*, downloaded from https://data.worldbank.org/indicator/SP.DYN.CDRT.IN) and the exposed population size (*P*, accessed from https://hub.worldpop.org/project/categories?id=3).

## Supplementary information

Supplementary Information

## Data Availability

The data produced and analysed for this study are available upon request. The Modular Earth Submodel System (MESSy) is continuously further developed and applied by a consortium of institutions. The usage of MESSy and access to the source code is licensed to all affiliates of institutions which are members of the MESSy Consortium. Institutions can become a member of the MESSy Consortium by signing the MESSy Memorandum of Understanding. More information can be found on the MESSy Consortium Website (www.messy-interface.org).

## Code availability

The Modular Earth Submodel System (MESSy) is continuously further developed and applied by a consortium of institutions. The usage of MESSy and access to the source code is licensed to all affiliates of institutions which are members of the MESSy Consortium. Institutions can become a member of the MESSy Consortium by signing the MESSy Memorandum of Understanding. More information can be found on the MESSy Consortium Website (www.messy-interface.org).
