# Supplementary material for: Widespread reduction of ozone extremes in storylines of future climate
Source: NPJ Clean Air. 2025 Aug 4;1(1):19. doi: 10.1038/s44407-025-00019-4 (PMC12321569; doi:10.1038/s44407-025-00019-4)
Supplement: Supplementary file 1 — Supplementary Information [file 44407_2025_19_MOESM1_ESM.pdf]

Supplementary Information

Widespread reduction of ozone extremes in storylines of future climate

Tamara Emmerichs[1,2], Domenico Taraborrelli[1,3*], Fuzhen Shen[4], Sergey Gromov[5], Michaela I. Hegglin[3,4,6,7] and Andreas Wahner[1]

[1]Institute of Climate and Energy Systems: Troposphere (ICE-3), Forschungszentrum Jülich GmbH, Jülich, Germany.
[2]now at Max-Planck Institute for Meteorology (MPI-M), Hamburg, Germany.
[3]Center for Advanced Simulation and Analytics (CASA), Forschungszentrum Jülich GmbH, Jülich, Germany.
[4]Institute of Climate and Energy Systems: Stratosphere (ICE-4), Forschungszentrum Jülich GmbH, Jülich, Germany.
[5]Max Planck Institute for Chemistry, Mainz, Germany.
[6]Department of Meteorology, University of Reading, Reading, United Kingdom.
[7]Institute for Atmospheric and Environmental Research, University of Wuppertal, Wuppertal, Germany.

*Corresponding author(s). E-mail(s): d.taraborrelli@fz-juelich.de; Contributing authors: t.emmerichs@fz-juelich.de; f.shen@fz-juelich.de; sergey.gromov@mpic.de; m.i.hegglin@fz-juelich.de; a.wahner@fz-juelich.de;

Check list:
This file includes:
Supplementary Table S1.
Supplementary Figures S1 to S12
Supplementary Note S1.

Table S1 Global budget of the O_x family [in Tg(O_3)/yr] for 2018. The standard deviation among the years is 1 % for the chemistry and dry deposition and 6 % for the residual. The O_x family is defined here as (molar) sum (O_3, $O(^1D)$, $O(^3P)$, NO_2, ClO, BrO, IO). The chemical production term accounts for all RO_2 + NO reactions producing NO_2. The chemical loss term accounts for $O(^1D)$ loss by water, O_3 reaction with HO_2 and OH, O_3 loss by halogen chemistry and reactions with VOCs.

Region	NH	SH	Global
Chemical production	3192	2039	5203
Chemical loss	2770	2018	4778
Dry deposition	515	265	780
Residual[1]	93	244	355
Burden	206	165	371

[1]The residual is the sum of the influx from the stratosphere and heterogeneous losses.

Supplementary Information Widespread reduction of ozone extremes in storylines of future

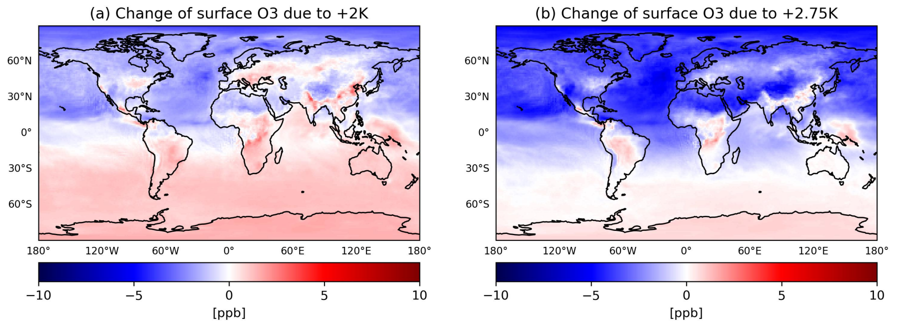

Fig. S1 Multi-year mean (2018-2020) absolute change of surface O_3 in two different climates climate at ground level in boreal summer. (a) +2K-*factual* climate and (b) +2.75K-*factual*.

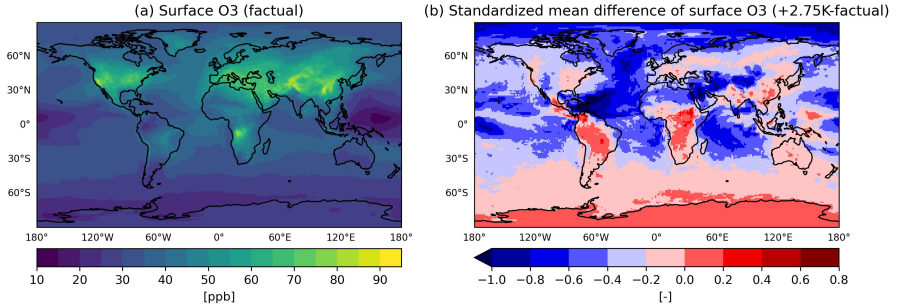

Fig. S2 Global distribution of (a) daily mean O_3 (factual, *+1.1K* vs. *pre-industrial*) and (b) the standaridized mean difference ($smd = \frac{mean(+2.75K)-mean(factual)}{sd(factual)}$) at ground level in summer 2018.

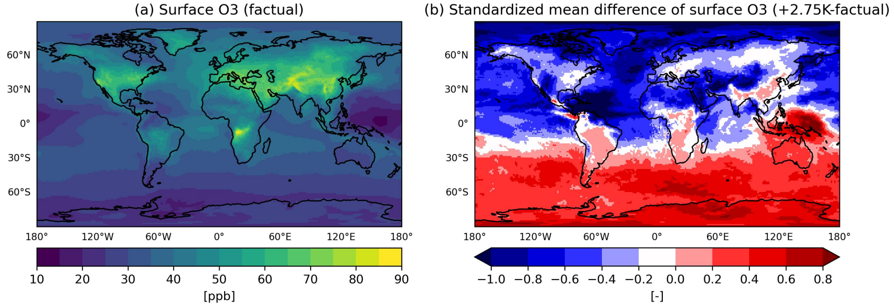

Fig. S3 Global distribution of (a) daily mean O_3 (*factual*, *+1.1K* vs. *pre-industrial*) and (b) the standaridized mean difference ($smd = \frac{mean(+2.75K)-mean(factual)}{sd(factual)}$) at ground level in summer 2019.

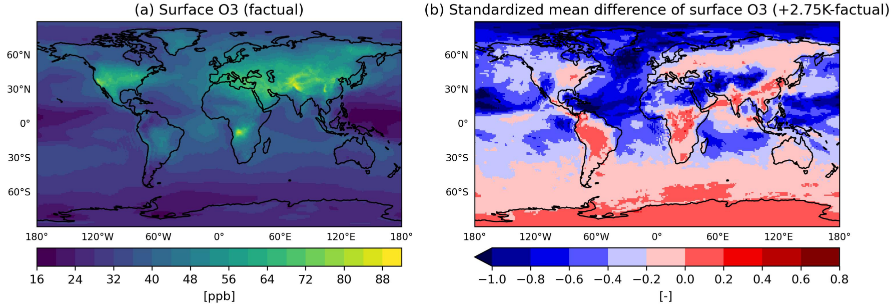

Fig. S4 Global distribution of (a) daily mean O_3 (factual, *+1.1K* vs. *pre-industrial*) and (b) the standaridized mean difference ($smd = \frac{mean(+2.75K)-mean(factual)}{sd(factual)}$) at ground level in summer 2020.

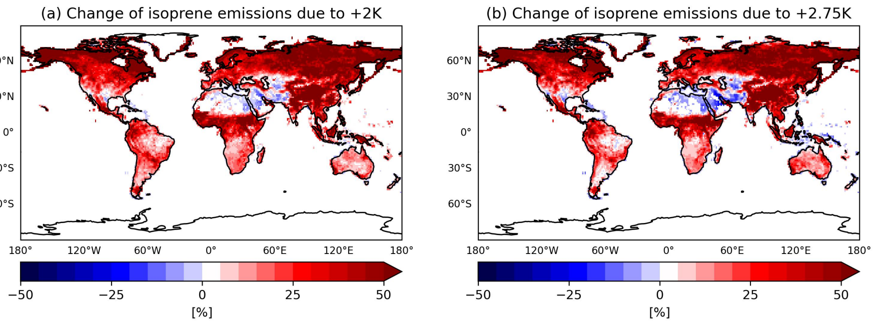

Fig. S5 Relative change of isoprene emissions (a) due to *+2K*, (b) and to *+2.75K* in boreal summer 2018.

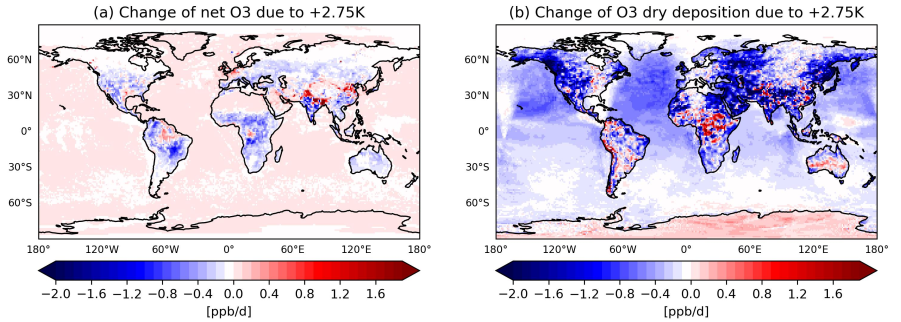

Fig. S6 Absolute change of the night-time (one day) accumulated (a) net O_3 chemistry at ground level and (b) the O_3 dry deposition due to the *+2.75K*-climate during summer (JJA) 2018.

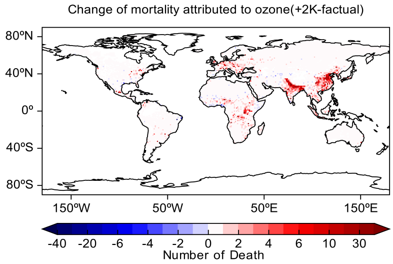

Fig. S7 Multi-year (2018-2020) mean premature mortality difference between *+2K* and the *factual* (, *+1.1K* vs. pre-industrial) climate. (Note: white color in color bar (a) means zero).

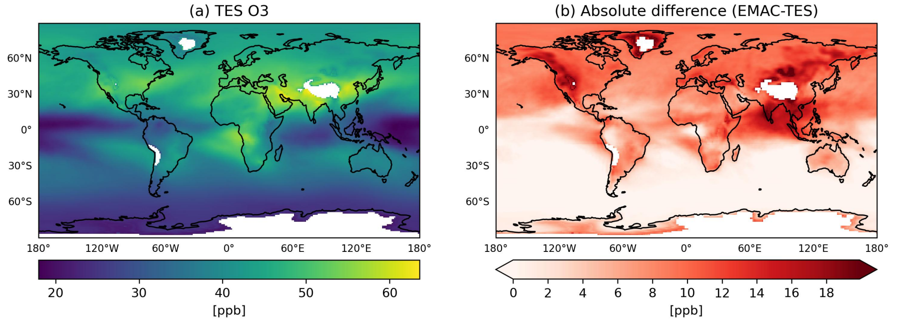

Fig. S8 Annual mean global distribution of mean tropospheric O_3 (700-800 hPa) of (a) the chemical reanalysis TES and (b) the difference to EMAC (factual-TES) in 2018.

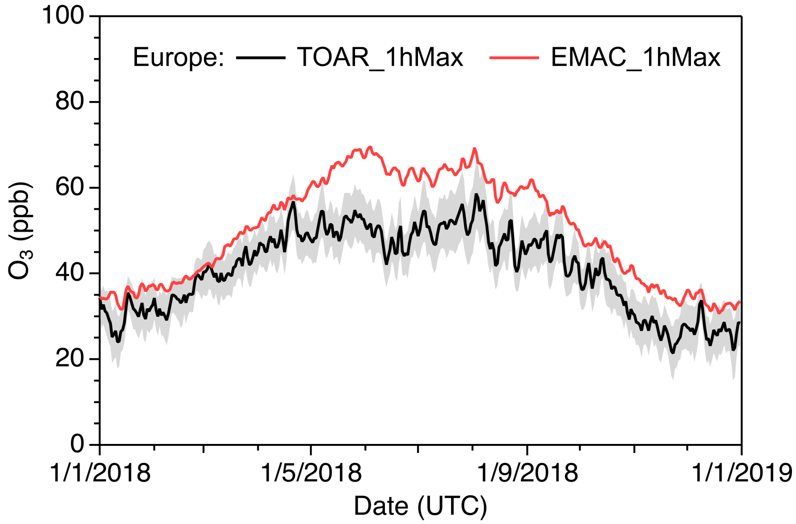

Fig. S9 Daily 1h-max O_3 in 2018 by EMAC compared to gridded TOAR observation data averaged across Europe. The gray shaded area represents the $\pm$ 1 σ of the observations.

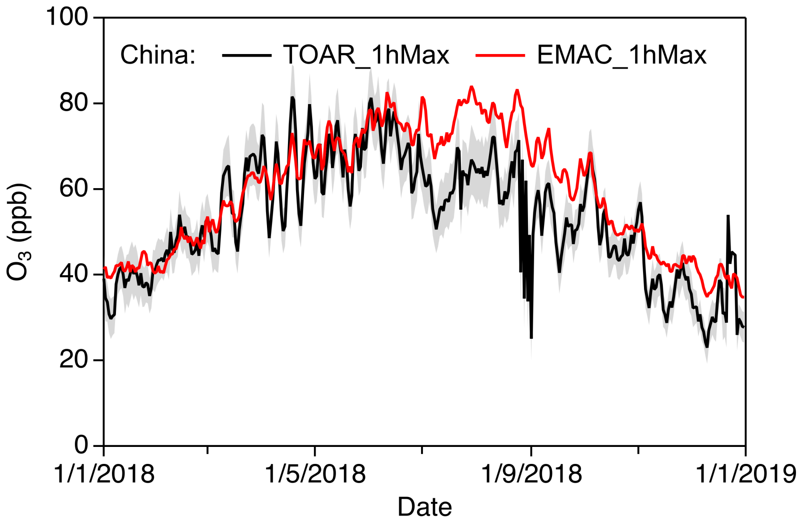

Fig. S10 Daily 1h-max O_3 in 2018 by EMAC compared to gridded TOAR observation data averaged across China. The gray shaded area represents the $\pm$ 1 σ of the observations.

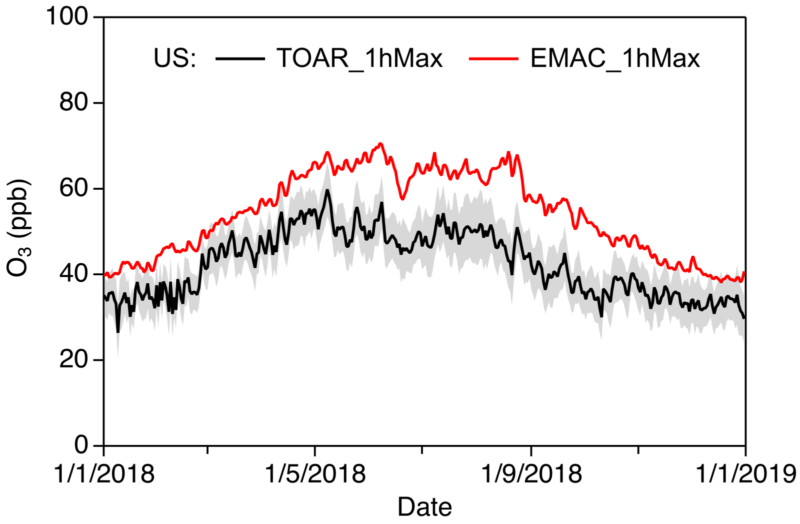

Fig. S11 Daily 1h-max O_3 in 2018 by EMAC compared to gridded TOAR observation data averaged across US. The gray shaded area represents the $\pm$ 1 σ of the observations.

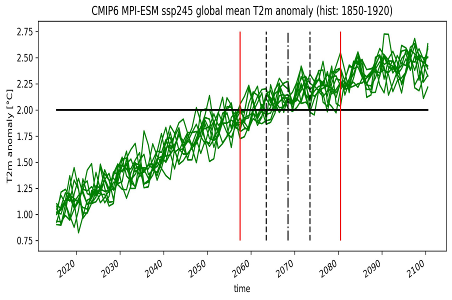

Fig. S12 Anomaly of 2m temperature by the first 10 ensemble members of the MPI-ESM simulations until 2100 compared to the reference period of 1850-1920.

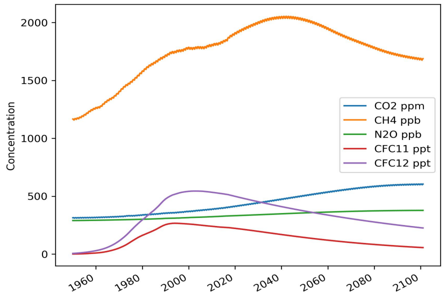

Fig. S13 Timeseries of global mean (weighted average) long-lived climate gases prescribed in EMAC .

S1 Warm spell representation in 2018-2020

Initiated by strongly positive blocking anomalies and a double-jet stream configuration over Europe the Northern Hemisphere and in particular Europe had 2018 the second warmest summer on record suffering from severe heat waves following an unusual warm and dry spring season. Different countries experienced multiple new records. In fact, Germany reported the periods April-July and the entire year as warmest on record while being the fourth driest year on record due to the prolonged drought from February to November. Similarly, UK and England reported the hottest summer since 1884. The Iberian Peninsula experienced a heatwave in particular in August which pushed the month on the top rank. In Finland, the summer temperature record since 40 years was broken by a newly measured value of 33 °C [1]. 2019 even experienced the warmest ever recorded throughout Europe while a short heatwave in July lead again to record-breaking temperatures throughout Europe [2]. The year 2020 is among the three warmest years on record where several widespread heatwaves and warm spells were recorded globally, in particular from June-August [3].

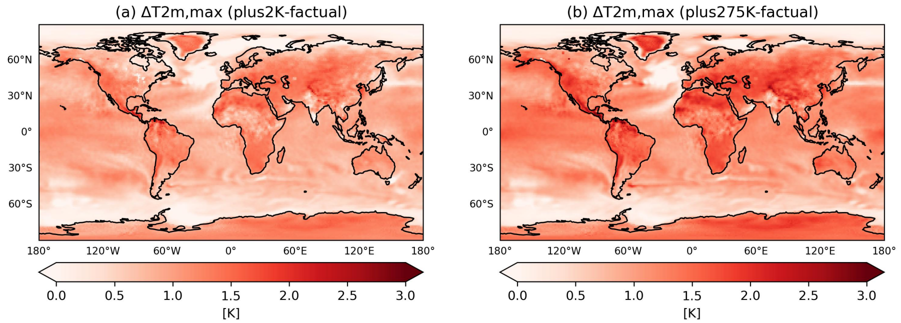

Fig. S14 Multi-year (2018-2020) summer (JJA) mean change of 2m temperature daily maximum (a) due to the *+2K* and (b) the *+2.75K* EMAC simulation.

Heating the Earth to *+2K* and *+2.75K* yields an annual global mean difference of +0.82 K and +1.06 K (+1.06 and +1.37K over land) relative to the 2018-2020 average, respectively. This confirms that the application of the storyline approach, warming the world in 2018-2020 by 2K (*+1.1K* vs. pre-industrial period) and *+2.75K*, is successful. Figure S14 shows a spatially uniform increase of the summer mean 2m temperature by +1-1.5 K in the *+2K*-climate and by +2-2.5K in the *+2.75K*-climate, on average. The minimum (over land) over India can be explained by the high aerosol concentration there which increase in the warmer climates and lead to an increased cooling effect. Because the sea surface temperature (SST) and sea ice content (SIC) is prescribed SST the storylines do not represent SST anomalies in the ocean and a realistic ice coverage in the Arctic [4]. Thus, the results over these areas cannot be interpreted.

Supplementary Information Widespread reduction of ozone extremes in storylines of future

References

[1] Rousi, E., Fink, A.H., Andersen, L.S., Becker, F.N., Beobide-Arsuaga, G., Breil, M., Cozzi, G., Heinke, J., Jach, L., Niermann, D., Petrovic, D., Richling, A., Riebold, J., Steidl, S., Suarez-Gutierrez, L., Tradowsky, J.S., Coumou, D., Düsterhus, A., Ellsäßer, F., Fragkoulidis, G., Gliksman, D., Handorf, D., Haustein, K., Kornhuber, K., Kunstmann, H., Pinto, J.G., Warrach-Sagi, K., Xoplaki, E.: The extremely hot and dry 2018 summer in central and northern europe from a multi-faceted weather and climate perspective **23**(5), 1699–1718. https://doi.org/10.5194/nhess-23-1699-2023. Accessed 2023-05-14

[2] Vautard, R., Aalst, M.v., Boucher, O., Drouin, A., Haustein, K., Kreienkamp, F., Oldenborgh, G.J.v., Otto, F.E.L., Ribes, A., Robin, Y., Schneider, M., Soubeyroux, J.-M., Stott, P., Seneviratne, S.I., Vogel, M.M., Wehner, M.: Human contribution to the record-breaking june and july 2019 heatwaves in western europe **15**(9), 094077. https://doi.org/10.1088/1748-9326/aba3d4. Accessed 2024-02-14

[3] Blunden, J., Boyer, T.: State of the climate in 2020 **102**(8), 1–475. https://doi.org/10.1175/2021BAMSStateoftheClimate.1. Accessed 2024-02-14

[4] Sánchez-Benítez, A., Goessling, H., Pithan, F., Semmler, T., Jung, T.: The july 2019 european heat wave in a warmer climate: Storyline scenarios with a coupled model using spectral nudging **35**(8), 2373–2390. https://doi.org/10.1175/JCLI-D-21-0573.1. Accessed 2022-04-06
